# The consultants’ role in the referring process with general practitioners: partners or adjudicators? a qualitative study

**DOI:** 10.1186/1471-2296-14-153

**Published:** 2013-10-11

**Authors:** Olav Thorsen, Miriam Hartveit, Anders Baerheim

**Affiliations:** 1 Department of Global Public Health and Primary Care, University of Bergen, Bergen, Norway; 2 Stavanger University Hospital, Stavanger, Norway; 3 Research Network of Integrated Care in Western Norway, Helse Fonna HF, Haugesund, Norway

## Abstract

**Background:**

Within the health system, communication between the different levels of care is essential for the patients’ clinical pathways and medical treatment. This includes the referral process: how and why patients are sent from the primary care level to specialist health services. We wanted to identify and describe hospital consultants’ reflections on and attitudes to the referral process and cooperation with general practitioners (GPs).

**Methods:**

A qualitative study of semi-structured interviews with 13 hospital consultants representing eight different specialties, analyzed using systematic text condensation. Interviews conducted from February 2011 to October 2012.

**Results:**

The consultants reported a considerable workload assessing referrals from GPs and prioritizing patients for specialist services. National guidelines were used as well as individual standards and guidelines. Good referrals could make the prioritization process easier. The specialists expressed a deep concern about securing a fair priority of patients and a willingness to give reasonable advice back to the referring GP when rejecting a referral. Better communication, such as a telephone call to confer with a hospital specialist before referral, was wanted.

**Conclusions:**

Better communication and cooperation between hospital consultants and GPs could make the referral process more balanced, and the participants more like partners.

## Background

The benefits of well-functioning primary care as the basis of a health system are abundant and consistent. Countries with health services based upon general practitioners (GPs) taking care of most medical problems of the population have both more equitable distribution of and more cost-effective health services [1]. Within the health system, communication between the different levels of care is essential for the patients’ clinical pathways and medical treatment. This includes the referral process: how and why patients are sent from the primary care level to specialist health services.

The referral system has a long tradition in many countries. Referral rates have been accelerating in many countries during the past decade, and the consequences are more use of specialist services and greater expenditure on health. The increasing referral rates and the reasons for these trends have been the subjects of many studies [2-7]. In the USA, from 1999 to 2009, the probability that an ambulatory visit to a physician would result in a referral to another physician increased by 94% from 4.8 to 9.3% [2]. The reasons for this situation are many, such as greater availability of specialty care, cultural changes, new national laws and regulations, more insecurity and uncertainty among GPs, especially the youngest, and patients’ increasing demands for specialist health services [3]. There are no internationally accepted guidelines for referral to specialists. The referral of patients is driven primarily by physician practice patterns [5]. The use of electronic referrals and online consultations accelerates the speed of communication and facilitates the logistics. This may reduce the need for patient–consultant meetings [6].

The consultants are the gate openers to the clinical pathways in hospitals [8]. When assessing referrals from GPs, either the consultants prioritize patients for further examination or treatment in specialist health services, or reject the referral. The referral process is composed of different stages, based on the sequence and purpose of events and tasks: (1) the consideration and decision to refer a patient to specialist health services; (2) the submission of the referral request and the referral review by the consultant; and (3) the patient transition into specialty care. The hospital consultants assess appropriateness, timeliness and completeness; a process that sometimes requires additional information. The referral can either be accepted and the patient given a scheduled appointment, rejected, or sometimes deferred for further discussion with another consultant. Acceptance triggers a series of steps to coordinate patient transition into the specialty setting, including communication with patients to schedule appointments, eventually followed by appointment reminders. Except for urgent cases, the GP’s recommendation or wish for specialist care within a certain time can be overruled by the consultant.

In 2011, we conducted a study on GPs’ thoughts and feelings about referral. This showed that GPs often felt humiliated and embarrassed, and had a wish for better communication and mutual understanding about the referral process [9]. In this study our aim was to investigate and identify hospital consultants’ reflections on and attitudes to the referral process and cooperation with the referring GPs.

## Methods

We conducted a qualitative study, based on semi-structured interviews. The first author interviewed a purposive sample of 13 experienced hospital consultants (two female and 11 male; aged 40–63 years) representing eight different specialties at Stavanger University Hospital, which covers 350,000 inhabitants in the South-western part of Norway. The specialists (three psychiatrists, one cardiologist, two orthopedic surgeons, two gynecologists, one pediatrician, one vascular surgeon, one gastroenterologist, and two general surgeons) were among those who received most referrals from GPs. We aimed for diversity in age, sex and specialties. In three of the interviews (orthopedics, gynecology, and general surgery) two consultants participated together. The hospital specialists were recruited until saturation was reached [10]. We used open questions about their work with and thoughts about the assessment of referrals from GPs, and how they used them to prioritize patients for further examinations and treatment in hospital. The 10 interviews lasted for 1 h each, and took place from February 2011 to October 2012. The first author informed the participating consultants about the study and conducted the interviews. All interviews were fully transcribed verbatim. The transcripts were analyzed by systematic text condensation [11]: (1) obtaining an overall impression; (2) identifying and sorting meaning units; (3) condensation – from code to meaning; and (4) synthesizing – into descriptions and concepts. At each of the four analytical steps, the three authors first analyzed the data individually and then contested each other’s analysis and reached a mutual basis for further analysis and final consensus.

## Results

### The workload of assessing referrals

All the 13 respondents stated that the workload of assessing referrals and prioritizing patients for further investigation and treatment was considerable. Some consultants required several hours per week, and sometimes a whole day. The number of referrals for assessment could rise to 150 per week. The time spent on each referral varied from 30 s to 10 min, depending on the case. Several said that they received many unnecessary referrals. All agreed on the importance of the quality of the referral on reducing the workload related to prioritizing patients.

*“We do an immense work in assessing referrals and to prioritize patients. Anything that can make this workload easier is positive!”* (Consultant 2)

If information was incomplete, it was important to determine the purpose of the referral to secure a fair prioritization. Incomplete referrals were not an acceptable reason for rejection. To reject a referral took more time than just accepting the patient onto the waiting list.

### The quality of referrals

All the respondents had specific ideas about what they wanted in a referral, and according to these, the referrals were described as good, insufficient or bad. Other descriptions of referrals were “vague” and “imprecise”, and the consultants were sometimes unsure as to whether the GPs themselves were aware of what they were asking. Some said that the referrals were generally not good; that they rarely received very good referrals and that many were insufficient and missing information about previous treatments, actual life situation, an accurate description of the symptoms and the patient’s motivation for treatment. It could also be difficult to discover the actual health problem and the severity of the case. Sometimes they observed a “cut and paste” from previous records, old notes and consultations that they had to scroll through to find the actual issue. Referrals should be more precise, to the point, and less cut and paste. The most important factor was to have a clear order, with symptoms and diagnoses, actual medication, and specific wishes.

*“We receive unnecessary referrals from GPs who are clearly not updated on particular issues. Many referrals are good and complete, but some are incomplete, especially from young doctors and doctors with different cultural backgrounds.”* (Consultant 6)

Some expressed personal opinions of referring physicians. One said:

*“You have a bunch of colleagues out there where you know the quality of referrals is bad, either really short or just an enumeration of the entire medical record, where you have to find out yourself. We know quite a few colleagues out there who refer very easily. We dislike that!”* (Consultant 8)

Several called for special templates for referrals that would contain mandatory information. Some had made their own guidelines and forms for special problems and diagnoses. Most referrals were sent electronically, and sometimes this made the referrals better, but they could still be more precise and accurate. Many stated that the GPs should do more before referring, for example X-rays or blood tests.

### The process of prioritizing

None of the consultants had any formal training in assessing referrals. It was something that they learned by themselves. The respondents said that the assessments and prioritizations could differ depending on who did it, but only the psychiatrists said that they consulted other colleagues when in doubt.

All respondents considered the assessment of referrals and prioritization of patients as important, and all emphasized the importance of precise referrals as essential for a reasonable and fair prioritization process. Many felt that the decisions meant a lot to the patients. The national guidelines for prioritization [12] should be followed. They were introduced to enable better prioritization; otherwise long waiting lists and the lack of finances and resources effectively reduced the capacity to accept all the patients who wanted a specialist assessment.

To give the patient the right priority it was important to obtain the correct interpretation of the referral. If in doubt, many of the respondents said that they rather wanted to see the patient instead of seeking supplementary information from the referring GP.

*“To assess a patient’s need for medical treatment is demanding, especially if you have to reject them. You have a person who you think needs help, but he does not fit the necessary criteria. So you have to reject, and this is a stressful job to do, to say no to someone. So the best thing is to get enough information.”* (Consultant 5)

If the consultant found that further investigation or treatment was unnecessary or contraindicated, they felt a responsibility to provide an oral or written explanation to the referring doctor. To reject a referral was not easy, and it was supposed to cause much discomfort to the patients. Not to be accepted could be embarrassing and humiliating for the patient, and this was sometimes a reason for the consultant to accept a referral that would normally be rejected. A rejection should be justified in a careful manner both to the patient and the referring GP. Several said that they owed to the patient and the GP that they did a thorough job. Some said that they always wrote a personal letter to the GP to justify the rejection of a referral, including a suggestion for further treatment or follow-up. The referring GP’s suggestion or wish for a maximum waiting time was overruled by the consultants’ prioritization of these patients.

### The relationship between consultants and GPs

All the interviewed consultants expressed the importance of good communication and cooperation with the referring GPs. One said that he felt like a judge with little experience. Many said that the GP should more often make a telephone call and confer with a hospital specialist before referring a patient. This was useful, but they did not experience that the GPs did this often.

*“One could avoid many referrals if the GPs called us and clarified the issues before referring.”* (Consultant 4)

The respondents said that they seldom contacted the GPs for additional information; mostly because this took time. It was discouraging when they were not able to get in touch with the GP on the telephone. Some were reluctant to call the referring GP if this could be interpreted as criticism. Several specified their role as consultants, and not as one taking over the total responsibility for treatment. At the same time they emphasized the GPs’ responsibility for the patients during the waiting time for specialist services.

## Discussion

Our findings confirm the importance of smooth and seamless cooperation in the referral process. All of the respondents reported a considerable workload assessing referrals from GPs and prioritizing patients for specialist services. This work was considered important in providing patients with a fair and reasonable waiting time for further investigations and treatment. The national guidelines were used, as well as individual standards and guidelines. Good referrals were said to make the prioritization process easier. The consultants expressed a deep concern about securing a fair priority of patients and a willingness to give reasonable advice back to the referring GP when rejecting a referral. Many referrals were regarded as unnecessary, meaning that the problem could be handled by the GP. Better communication, such as a telephone call to confer with a hospital specialist before referring was wanted, and could possibly reduce the referral rates.

There are numerous studies on consultants’ evaluation of the quality of referrals [13-18]. In most of these, the hospital specialists reported many inadequate and unnecessary referrals. Our respondents shared this opinion. The consultants’ wished for specific forms or templates designed for the different medical conditions and diagnoses, and believed these would make this work easier and smoother. The Norwegian national guidelines for prioritization [12] were introduced in 2012, to help the hospital consultants to choose the right patients for specialist care, and to ensure that patients have a fair and equal assessment, regardless to which hospital they are referred. They also indicate maximum waiting times for treatment according to the different conditions and diseases. In our study, half of the respondents used these guidelines while prioritizing. Such guidelines could be used automatically to sort and prioritize patients for specialist care. According to CB Forrest, “*The absence of clarity in the specialist physician clinical role makes it unlikely that specialists are being used effectively and efficiently. We lack agreement on the core clinical functions of health care specialism, when patients should be referred to specialists, and how long specialists should be involved in a referral. This uncertainty is a likely contributor to the marked variation in the use of specialty care across the country*” [19]. In our study, the interviewed consultants expressed a deep concern making sure the process was fair and equal for the patients. When refusing a patient for medical examination or treatment, they emphasized the responsibility for explaining this to the referring GP, and eventually giving advice for alternative handling or treatment.

The respondents had extensive experience, but no formal education or training in assessing referrals. They confirmed that there was a risk of inequity and unfair prioritization, but only the psychiatrists conferred with other colleagues when in doubt. A recent study confirms this danger [20]. National guidelines for prioritizing patients may prevent some of this source of error.

The professional relationship between consultants and the referring GPs has been described in several articles [5-7,9,11]. In our study the respondents did not agree on having a judgmental role, but confirmed the quality assessment task of the referrals and the power to prioritize patients for specialist care and eventually to reject the referral. Most of the respondents expressed a willingness to see the patient when in doubt. Long waiting lists may influence this attitude, as well as personal connections and relationships, leading to injustice and inequality. The referring GPs’ suggestion or wish for a maximum waiting time was overruled by the consultants’ prioritization of these patients, which puts the consultant in a superior position versus the referring colleague. This confirms the GPs’ feelings of an inferior role in this process [9]. The concept of shared care was not mentioned by the respondents. Both GPs and consultants want an easier and smoother communication about difficult medical problems, by telephone, e-mail or personal contact, before and eventually instead of a referral [5-7,9,10,21-23].

The referral patterns are important focal points of both politicians and health managers to control health care spending [1-3,21-25]. Even in countries without this tradition, such as China, the advantages of a referral system are of interest [26]. GPs want more shared care for their patients [9]. Both consultants and GPs express a “them and us” attitude, more than “we as a team”. Legislative and structural regulations as well as personal relationship and mutual respect are important factors in developing more collaboration [5-7,9,14].

In our study the interviewer was known to the respondents as an experienced GP and a researcher on the referring process. It is uncertain whether this has biased the statements and comments of the consultants. Three of the interviews were done in 2011, before the introduction of the national guidelines for prioritization. The impact on the respondents’ statements about this may be important. In the analyzing process the other two co-authors have done their own individual analyses, securing a balanced consensus of the results and conclusions. The majority of male respondents may be a possible source of error. In the citations the respondents were given a number to ensure anonymity.

## Conclusions

Better communication and cooperation between hospital consultants and GPs could make the referral process more balanced, and the participants more like partners. New models for collaboration should be tried out.

## Competing interests

The authors declare that they have no competing interests.

## Authors’ contributions

OT designed the study, led the analysis of the data and led the writing. MH analyzed the data and contributed to the writing. AB designed the study, conducted the research, analyzed the data and contributed to the writing. All authors have read and approved the final manuscript.

## Pre-publication history

The pre-publication history for this paper can be accessed here:

http://www.biomedcentral.com/1471-2296/14/153/prepub

## References

[B1] Starfield B Shi L Macinko J Contribution of primary care to health systems and health Milbank Q 2005 83 457 502 10.1111/j.1468-0009.2005.00409.x 16202000 PMC2690145

[B2] Barnett ML Song Z Landon BE Trends in physician referrals in the United States, 1999–2009 Arch Intern Med 2012 172 163 170 10.1001/archinternmed.2011.722 22271124 PMC3568395

[B3] Forrest CB Nutting PA von Schrader S Rohde C Starfield B Primary care physician specialty referral decision making: patient, physician, and health care system determinants Med Decis Making 2006 26 76 85 10.1177/0272989X05284110 16495203

[B4] Katz MH How can we know so little about physician referrals? Arch Intern Med 2012 172 100 10.1001/archinternmed.2011.1290 22271117

[B5] Manca DP Breault L Wishart P A tale of two cultures: specialists and generalists sharing the load Can Fam Physician 2011 57 576 584 21642740 PMC3093596

[B6] Gandhi T Sittig DF Franklin M Sussman AJ Fairchild DG Bates DW Communication breakdown in the outpatient referral process J Gen Intern Med 2000 15 626 631 10.1046/j.1525-1497.2000.91119.x 11029676 PMC1495590

[B7] Fulton J The both of us Can Fam Physician 2011 57 525 21571708 PMC3093574

[B8] Akbari A Mayhew A Al-Alawi MA Grimshaw J Winkens R Glidewell E Pritchard C Thomas R Fraser C Interventions to improve outpatient referrals from primary care two secondary care Cochrane Database Syst Rev 2005 4 Art. No.: CD005471 10.1002/14651858.CD005471.pub2 PMC4164370 18843691

[B9] Thorsen O Hartveit M Baerheim A General practitioners’ reflections on referring: An asymmetric or non-dialogical process? Scand J Prim Health Care 2012 40 241 246 23050793 10.3109/02813432.2012.711190 PMC3520419

[B10] Kuzel AJ Crabtree BF, Miller WL Sampling in qualitative inquiry Doing Qualitative Research 1999 Thousand Oaks, CA: Sage Publications 33 45

[B11] Malterud K Systematic text condensation: A strategy for qualitative analysis Scand J Pub Health 2012 40 795 805 10.1177/1403494812465030 23221918

[B12] Forskrift om prioritering av helsetjenester, rett til nødvendig helsehjelp fra spesialisthelsetjenesten, rett til behandling i utlandet og om klagenemnd (prioriteringsforskriften). (Regulations to prioritize health services, the right to necessary care from specialist health services, the right to have treatment abroad and to appeal) Lovdata 2000 http://www.lovdata.no/for/sf/ho/xo-20001201-1208.html

[B13] Chen AH Yee HF Jr Improving primary care-specialty care communication: lessons from San Francisco's safety net Arch Intern Med 2011 171 65 67 10.1001/archinternmed.2010.484 21220663

[B14] Jiwa M Arnet H Bulsara M Ee HC Harwood A What is the importance of the referral letter in the patient journey? A pilot survey in Western Australia Qual Prim Care 2009 17 31 36 19281672

[B15] Berendsen AJ Benneker WHGM Meyboom-de Jong B Klazinga NS Schuling J Motives and preferences of general practitioners for new collaboration models with medical specialists: a qualitative study BMC Health Serv Res 2007 7 4 10.1186/1472-6963-7-4 17207278 PMC1774564

[B16] Patel NN D’Souza J Rocker M Townsend E Morris-Stiff G Manimaran M Magee TR Galland RB Lewis MH Prioritisation of vascular outpatient appointments cannot be based on referral letters alone Surgeon 2008 3 140 143 18581748 10.1016/s1479-666x(08)80108-4

[B17] Bodek S Ghori K Edelstein M Reed A MacFadyen RJ Contemporary referral of patients from community care to cardiology lack diagnostic and clinical detail Int J Clin Pract 2006 60 595 601 10.1111/j.1368-5031.2006.00902.x 16700861

[B18] Blundell N Taylor-Phillips S Spitzer D Martin S Forde I Clarke A Elective surgical referral guidelines – background educational material or essential shared decision making tool? A survey of GPs in England BMC Fam Pract 2011 12 92 10.1186/1471-2296-12-92 21878103 PMC3176475

[B19] Forrest CB A typology of specialists’ clinical roles Arch Intern Med 2009 169 1062 1068 19506176 10.1001/archinternmed.2009.114

[B20] Holman PA Ruud T Grepperud S Horizontal equity and mental health care: a study of priority ratings by clinicians and teams at outpatient clinics BMC Health S Res 2012 12 162 10.1186/1472-6963-12-162 PMC3430583 22704131

[B21] McBride D Hardoon S Walters K Gilmour S Raine R Explaining variation in referral from primary to secondary care: cohort study BMJ 2010 341 c6267 10.1136/bmj.c6267 21118873 PMC2995017

[B22] Chen AH Yee HF Improving the primary care – specialty care interface. Getting from here to there Ach Intern Med 2009 169 1024 1026 10.1001/archinternmed.2009.140 19506170

[B23] Rittenhouse DR Shortell SM Fisher ES Primary care and accountable care – two essential elements of delivery-system reform N Eng J Med 2009 361 2301 2303 10.1056/NEJMp0909327 19864649

[B24] Mehrotra A Forrest CB Lin CY Dropping the baton: specialty referrals in the United States Milbank Q 2011 89 39 68 10.1111/j.1468-0009.2011.00619.x 21418312 PMC3160594

[B25] Rosemann T Wensing M Rueter G Szecseny J Referrals from general practice to consultants in Germany: If the GP is the initiator, patients’ experiences are more positive BMC Health S Res 2006 6 5 10.1186/1472-6963-6-5 PMC1386652 16423299

[B26] Du J Lu X Wang Y Cui S Guo A Coid D Wang W Mutual referral: a survey of GPs in Beijing Fam Pract 2012 29 441 447 10.1093/fampra/cmr122 22223744

